# Extradigital glomus tumor of the anterior knee

**DOI:** 10.1080/23320885.2020.1810579

**Published:** 2020-08-29

**Authors:** Benjamin P. Christian

**Affiliations:** Division of Plastic Surgery, Brigham and Women’s Hospital, Boston, MA, USA

**Keywords:** Glomus, extradigital, Knee pain

## Abstract

The benign glomus tumor is an uncommon cause of crippling pain most commonly associated with the fingers. This sheep in wolf’s clothing is identified by careful examination, confirmed by MRI, and often resolved with a simple procedure. Here we present a patient with chronic knee pain of 21 years duration.

## Introduction

The glomus tumor arises from the glomus body which is broadly found in the dermis, and most densely in that of the digits. The glomus body participates in thermoregulation, acting as a blood flow shunt to alternatively reduce and promote convective heat loss. The glomus tumor is near universally benign though it’s hallmark is intense pain in the subungual location. We present the case of an extradigital glomus tumor causing chronic debilitating pinpoint pain in the anterior knee region which was fully alleviated with simple excision.

## Case report

A sixty-seven-year-old male diabetic with psoriatic arthritis presented to the plastic surgery clinic with an exquisitely painful ‘bump’ below the left knee first noticed several months earlier without clear etiology. Though the contour irregularity was new, his intense pain and hyperesthesia at this location seemed to develop shortly after repair of a ruptured quadriceps tendon 21 years previously. He had been plagued with pain in this region since then and learned to adjust most activities and avoid even the lightest touches, like a bedsheet. Multiple prior imaging studies including plain radiographs and MRIs over this lengthy period were unrevealing for the causative etiology. Despite the tenderness, he had no pain with ambulation, joint loading, or full knee motion. He denied overlying skin changes or concern for infection.

Inspection of the left knee region revealed a mature vertical midline scar over the distal thigh and patella and a visible 1.5 cm mass approximately 5 cm distal to the inferior patellar margin without overlying skin changes. Attempts at examination were met with repeated quick jerking withdrawals. With time, he was found to have hyperesthetic skin over the entire patella and was exquisitely tender over the pretibial lesion. Pain was not reproduced with knee or distal extremity passive or resisted active motion. Gait was normal.

Office-based ultrasound demonstrated a well-circumscribed homogenous echogenically gray nodule 10 × 15 × 5mm superficial to the patellar tendon. With Doppler imaging, it was extremely vascular with a strong pulsatile signal throughout the entire nodule. Subsequent MRI revealed an ovoid 4 × 13 × 8mm T2 hyperintense homogenously enchancing lesion with mild adjacent subcutaneous fat edema overlying the tibial tubercle.

The patient underwent excisional biopsy of the mass under IV sedation. He was too anxious to attempt excision under local anesthesia and would almost involuntarily recoil with any attempt to approach the knee region. The surrounding tissues were infiltrated with epinephrine-containing local anesthetic and no tourniquet use was employed. Sharp dissection through the superficial soft tissue revealed a well-circumscribed deep purple mass overlying the patellar tendon. The mass was easily dissected free from the tendon and removed with 3.5x loupe magnification. Pathology confirmed the suspected glomus tumor and the diagnosis was supported by immunohistochemical studies that show positive staining for smooth muscle actin and negative staining for desmin, pancytokeratin, and S-100 (2). Furthermore, the lesion appeared well circumscribed and was without appreciable atypia or mitotic activity.

**Figure 1.**
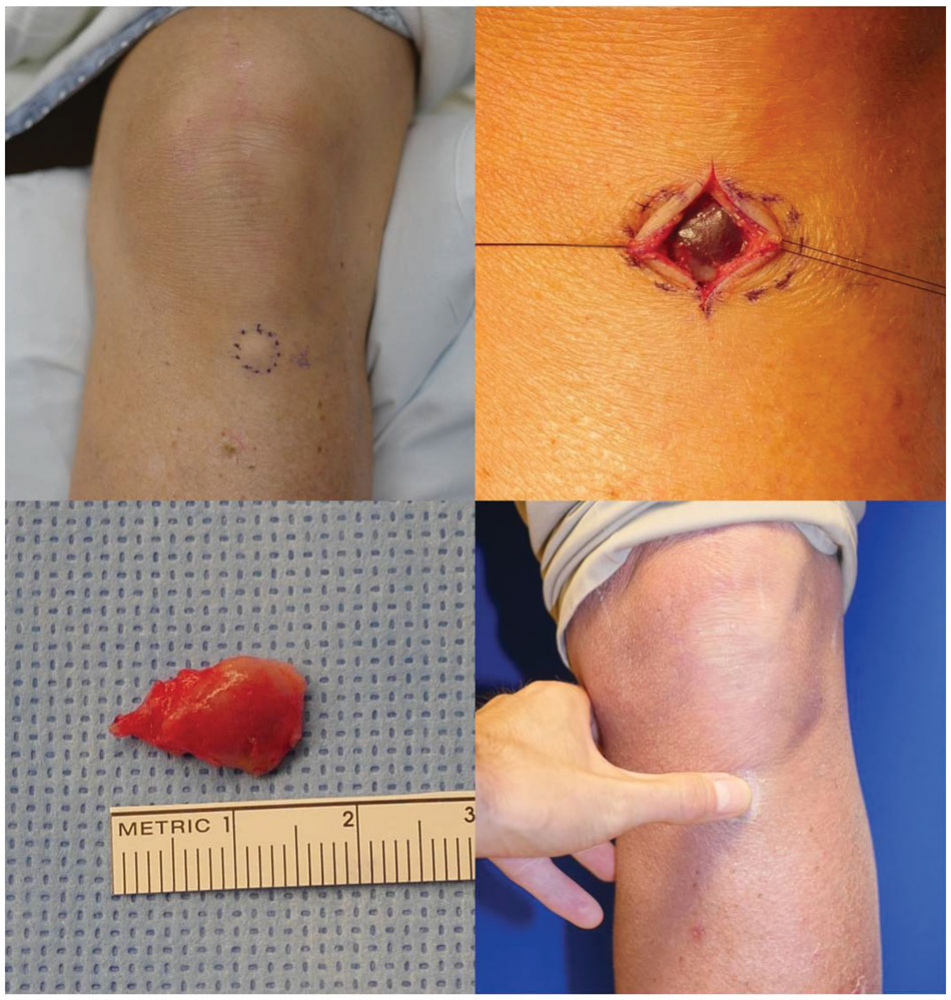
Pre-operative knee with tumor outlined, *in situ* and resected tumor, post-operative tolerance of pressure over prior tumor site and surgical scar.

**Figure 2.**
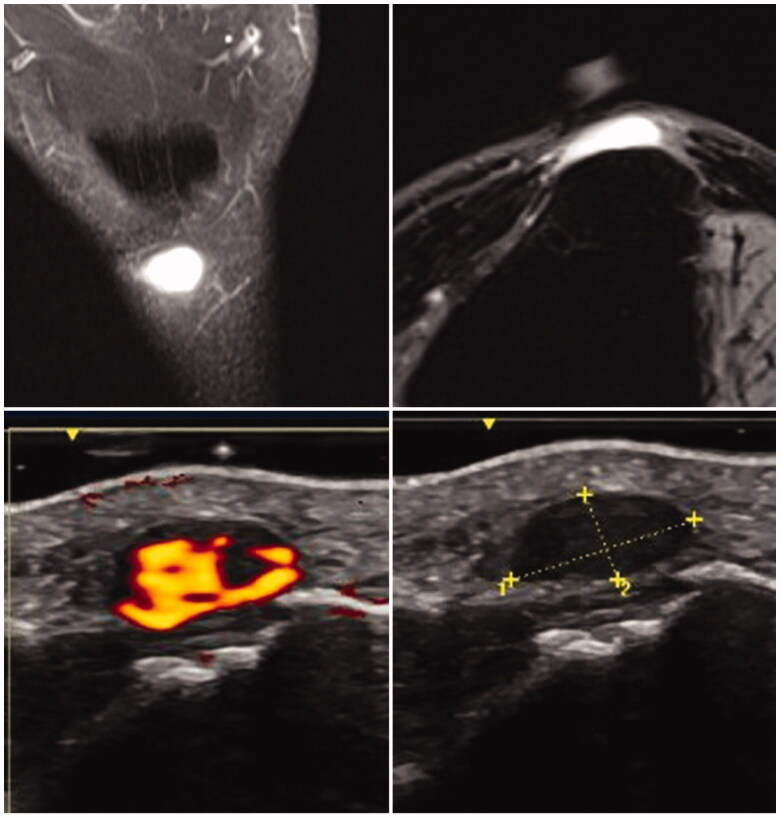
Top images from pre-operative MRI. T1 fat saturated in coronal and axial planes. Bottom images from pre-operative doppler ultrasound.

At the patient’s first post-operative office visit, he was completely free of pain and tenderness in the entire knee region and easily tolerated direct pressure to the area. After nearly three years, he continues to be free from pain (Figures 1 and 2).

## Discussion

The glomus body is a neuro-myo-arterial apparatus in the subcutis and reticular dermis which participates in thermoregulatory control. It is composed of an arteriole and venule with multiple shunts, perivascular contractile glomus cells, and non-myelinated nerve fibers which influence vessel diameter through sympathetic tone [1]. In cold conditions, the glomus body assists in shunting blood away from the papillary dermis and skin surface, thereby decreasing heat loss from the body [2]. The converse is true for heat release or in conditions which provoke a flushing response.

A glomus *tumor* is a benign lesion arising from the glomus body which usually presents in the distal upper or lower extremities of young adults. They have a predilection for the subungual region of fingers, corresponding to one of the densest collections of glomus bodies. Although the hand is commonly affected, cutaneous lesions can occur at other sites, and extracutaneous glomus tumors have been reported in the gastrointestinal tract, bone, mediastinum, trachea, mesentery, cervix, and vagina among other disparate anatomic sites [1,3]. In the subungual location, they are more common in women whereas extradigital glomus tumors are more common in men 4:1 [1]. The tumor is generally a 5 mm red-bluish macule found within the nail bed [4]. In extradigital locations, the glomus tumor is a similarly-pigmented nodule and typically less than 2 cm in diameter. Overall these are rare tumors, and account for between 1 and 5% of upper extremity neoplasms, 75% of which occur in the hand and 50% in a subungual location [5]. Though most commonly thought of as idiopathic some studies suggest an association with trauma or injury to the area prior to the onset of symptoms [1].

Patients with glomus tumors classically present with a triad of pinpoint tenderness, paroxysmal pain, and cold hypersensitivity. Localization of the lesion can be performed with Love’s test by applying pressure to the suspected area with the head of a pin or paperclip, eliciting intense pain. Love’s pin test has a sensitivity of 100% and accuracy of 78% [6]. If a tourniquet or blood pressure cuff is utilized proximal to the lesion in Hildreth’s test, the tenderness is abolished. Hildreth’s test has a sensitivity of 71% and specificity of 100% with 78% accuracy [6]. Cold sensitivity was found to be 100% sensitive, specific, and accurate [6]. Subjective symptoms typically exceed clinical signs and it is not uncommon for patients to see multiple providers, sometimes over multiple years, prior to obtaining the correct diagnosis. Patients have been wrongly referred to psychiatrists due to misdiagnosed glomus tumor where no nail or skin alteration was visible, and no proper workup performed. Clinically, glomus tumors can be confused with neuromas, arthritis, gout, eccrine spiradenomas, and leiomyomas which can all be readily distinguished from glomus tumors histologically and immunohistochemically [4].

MRI is the gold standard imaging technique for identifying and characterizing glomus tumors which have been described as appearing dark and well-defined on T1-weighted images and bright on T2-weighted images with diffuse enhancement after gadolinium administration [7,8]. This tumor is hyper-vascular so it may be slightly brighter on T1 fat saturated images and will be bright on any fluid-sensitive sequence. MRI sensitivity approaches 90% whereas the specificity is estimated at 50% [9]. False-negatives occur particularly with small lesions measuring 2–3mm so the surgeon should proceed with exploration and excision if clinical suspicion is high despite a negative MRI [1].

Doppler ultrasound can be used to help detect the high blood flow associated with these lesions with a sensitivity approaching 90% [7]. Radiographs are of limited usefulness in diagnosis, but subungual tumors may cause bony erosion and may show increased distance between the nail and the dorsum of the phalanx [7]. Angiography, thermomography, and scintigraphy have all been investigated but play little role in standard investigations of these tumors [9].

Complete excision is generally curative, with resolution of all symptoms. In the digit, a tourniquet is typically used to facilitate dissection.

Glomus tumors are generally well-circumscribed sheets and clusters of uniform glomus cells around capillary-sized vessels. A pseudocapsule may be present. The glomus cells express vimentin and muscle actin isoforms. These tumors typically show no significant atypia but malignant degeneration has been described.

## Conclusion

Glomus tumors are benign lesions most commonly associated with crippling pain in the digits though they often occur in extradigital locations. In fact, over a 20 year period 61% of glomus tumors at one institution were extradigital [1]. Patients with glomus tumors commonly suffer a delay in diagnosis irrespective of tumor location, though extradigital locations may make the diagnosis even more challenging. MRI is the imaging modality of choice though diagnosis and surgical plan can be executed with a careful history and physical alone. These tumors are relatively easy to excise given their frequent encapsulation and superficial location. Careful and complete excision can deliver permanent relief of chronic and severe pain in a simple outpatient procedure. Persistence or recurrence of symptoms could be due to inadequate resection or unrecognized synchronous satellite lesions [2], but a symptomatic neuroma must also be considered [10].
